# Supplementary material for: Pharmacogenomics at the Point of Care: A Community Pharmacy Project in British Columbia
Source: J Pers Med. 2020 Dec 24;11(1):11. doi: 10.3390/jpm11010011 (PMC7823931; doi:10.3390/jpm11010011)
Supplement: Supplementary file 1 [file jpm-11-00011-s001.zip › Community_PGx_Supplement/Expanded_Online_Methods.pdf]

Expanded Methods

1.1. Pharmacy Selection

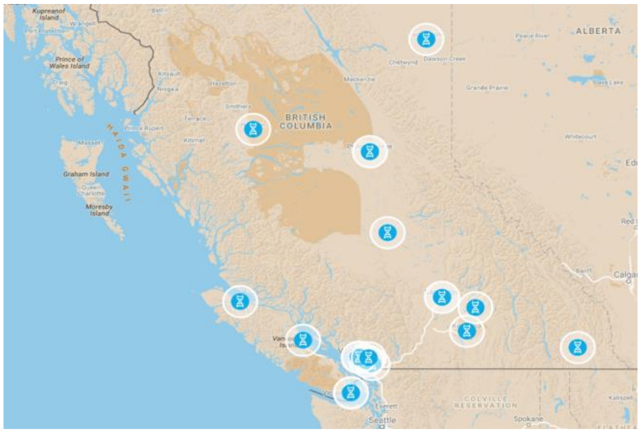

Figure S1: Map of participating pharmacies by their locations.

Pharmacy	City
Armstrong Pharmacy	Armstrong
BC Pharmacy Association	Kamloops
Harbourside Pharmachoice	Port McNeill
Hart Drug Mart	Prince George
Heart Pharmacy Ida	Victoria
Lakeside Medicine Centre	Kelowna
London Drugs #67	Courtenay
Naz's Pharmacy No. 2 Ltd.	Surrey
Pharmasave #032	Houston
Phoenix Dispensary	Prince George
Pratt's Compounding Pharmacy	Kamloops
Quadra Village Drug Mart	Victoria
Safeway #4918 (Willowbrook)	Langley
Save-on-foods Pharmacy #987	Williams Lake
Shoppers Drug Mart #2212	Surrey
Wellness Pharmacy #1	Vancouver
Wilson Pharmacy	Port Coquitlam

Table S1: Table of participating pharmacies and their locations.

Community pharmacies were selected to reflect a diversity in geography and practice environments in BC, Figure S1. Pharmacies were required to have expressed interest in participating, a corporate membership with the BC Pharmacy Association, a sufficiently private counselling area and adequate staffing to ensure that the pharmacist could have uninterrupted time with participants during the education and consent process. Additional pharmacies were added throughout the project as needed. At the start of this study, we had recruited 34 pharmacists at 21 different community pharmacies in 15 different communities. Taking into account individual turnover, we ended up with 21 pharmacists recruiting patients at 17 participating community pharmacies in 13 locales across the province as shown in Table S1.

1.2. *Pharmacist Training*

In addition to the Tri-Council Policy Statement Ethical Conduct for Research Involving Humans Course on Research Ethics the pharmacists had to complete a study training program done remotely via webinar and phone. The goals of the training were two-fold, i) to ensure pharmacists followed all the requirements of the law and Research Ethics Board of the University of British Columbia (UBC), especially with respect to patient privacy; and ii) to ensure that the patient experience was consistent regardless of the pharmacy type or location.

1.3. Privacy & Informed Consent

A study team member and the pharmacist discussed the project principles of informed consent, privacy requirements, patient education, obtaining consent, collecting patient information, and reviewed a consent checklist designed to guide the education and consent process. At the conclusion of this session, the pharmacist was asked a series of questions based on the training they received.

1.4. Operations Logistics and Report Interpretation

The details of sample collection, handling, return, and documentation were discussed with a team leader. Pharmacists were required to complete the myDNA online pharmacist

training program for PGx as well, providing an overview of pharmacogenomics as well as interpretation of the myDNA reports. The learning objectives for this training were 1) understand the basis of cytochrome (CYP) P450 genes/enzymes associated with CPIC guidelines, 2) understand how variants affect an individual's ability to metabolise medications, and 3) how to apply this knowledge in clinical practice to improve their patients' outcome.

1.5. Quality Control (QC)

Before the pharmacists enrolled patients in our study, a phone call to role play the registration and consent process with a study team member was conducted. The study team member completed the consent checklist (Supplement I) during the process and at the end of the session reviewed the terminology, phrasing, and content with the pharmacist.

1.6. Participant Selection

To be enrolled in the study a potential participant must have been over 19, speak English, and needed to be taking at least 1 of the mental health drugs listed in Table S2. Pharmacists were prohibited to search patient records to identify eligible participants.

Antidepressant	Antidepressant	Antipsychotic
Agomelatine	Mianserin	Aripiprazole
Amitriptyline	Mirtazapine	Clozapine
Citalopram	Moclobemide	Haloperidol
Clomipramine	Nortriptyline	Olanzapine
Dothiepin	Paroxetine	Quetiapine
Duloxetine	Sertraline	Risperidone
Escitalopram	Trimipramine	Zuclopenthixol
Fluoxetine	Vanlafaxine	
Fluvoxamine	Vortioxetine	
Imipramine		

Table S2: Table of eligible metal health medications.

1.7. Consent

The patient consent and enrollment process, like the pharmacist training, was rigorous and uniform, regardless of location or the pharmacist. A participant information session took approximately 30-45 minutes and proceeded as follows. In a private area of the pharmacy, the pharmacist explained the project and summarized the Participant Information & Consent Form (Supplement II). A checklist was completed for each potential participant. The potential participant was then shown a video specifically developed for this project. The video, (Supplement III-IV), introduced the key concepts of PGx and the goals of the research project. The pharmacist watched the video with each patient to ensure that concepts were clear and to answer questions as necessary. The potential participant was then given the Patient Information & Consent Form to review, and was required to wait at least 24 hours before committing to the study. This allowed patients time to reflect, to discuss the project with other family members or caregivers, and to obtain additional information to make an informed decision about their participation.

After a potential participant agreed to the study, the enrollment process took approximately 30-60 minutes. It started with the pharmacist answering questions generated in the contemplative (take-home) phase. Next, patients signed the consent form and were given a copy for their records. Following their consent, the patients provided a saliva sample and their pharmacist collected the required enrollment information. To avoid external incentives (or the appearance thereof) we specified that each pharmacist be limited to recruiting a maximum of 10 patients.

1.8. Data & Sample Collection

Mandatory information collected included date of birth, gender, current medications, history of ADRs as well as allergies and medical conditions. Optional fields included ethnicity, height, weight, and smoking history. Given the known impact of ethnicity on allele frequencies, as well as smoking's effect on drug metabolism, both should be mandatory in the future. The Genotek Oragene saliva collection kit was used according to manufacturer's protocol to collect

patient sputum [1]. This process took 2-5 minutes in most cases, although there were participants who took longer and a small number who were unable to provide usable saliva samples. The reasons for this varied, but the common theme was that these participants complained of 'dry mouth'. Although it was beyond the scope of this study, we expect that alternative collection methods (i.e. buccal swab) would have been a suitable alternative.

1.9. Experience Survey

A pharmacist and patient experience survey were mailed out with the recruitment kit (Supplement V-VI). The enrolling pharmacist ensured completion and return of the surveys at the end of the study. They were asked to indicate their level of agreement using a 4-point Likert scale, which was chosen over a 5-point scale. The 4-point Likert removes the "Neutral" option to require respondents to either agree or disagree with the statements [2].

1.10. Transport of Samples & Participant Information

After de-identification, the original copy of the patient enrollment documentation and the patient's saliva sample were sent via secure courier to UBC. A copy of the demographic information was kept and secured at the pharmacies. Saliva samples were received and catalogued and stored at our sequencing facility (https://sequencing.ubc.ca/). Participant information was used to update a key file linking identifying information to the participant code. All non-identifying information was transcribed and linked only to the participant code. Sample IDs were then subsequently linked to unique, randomized sample barcodes for downstream analysis and report tracking.

1.11. Sample Processing

DNA was extracted from 250 µl of saliva sample. Any remaining saliva was stored at room temperature for up to a week prior to long term storage at - 20 °C. The "prepIT.L2P" reagents were used according to the manufacturer's instructions (DNA Genotek). DNA was eluted in 50 µl molecular-grade water and DNA quality was assessed by gel electrophoresis and quantified by Nanodrop (Thermofisher Scientific - Waltham, MA) and fluorometry using the Qubit dsDNA HS Assay Kit. The gel analysis provided a go/no-go step for the samples, in other words, if samples were extensively degraded at this QC step, we attempted a second extraction. DNA was stored at -20 °C until genotyping or TRS library preparation.

1.12. TargetRich Sequencing (TRS)

DNA was extracted as described above and processed according to the manufacturer (https://www.kailosgenetics.com/). Briefly, to prepare the sequencing library, guide oligos which contain the sequences to be amplified are annealed, followed by a restriction enzyme digestion, after which Illumina adapter sequences are annealed along with the unique identifier (barcode) for the library sample. The samples are then enzymatically cleaned via magnetic beads before being amplified and cleaned a final time. Samples were QC'd by agarose gel electrophoresis and quantified with Qubit. Pooled amplicons were sequenced on an Illumina Miseq platform, generating paired-end 78 bp reads [3]. Long range PCR was used to determine duplication as described [4].

1.13. Genotyping

We worked with myDNA https://www.mydna.life/en-ca/ to perform SNP analysis using the iPLEX MassArray System, a non-fluorescent platform utilizing MALDI-TOF (matrix-assisted laser desorption/ionization — time of flight) mass spectrometry, coupled with end point PCR to measure PCR-derived amplicons in multiplexed reactions. Briefly, polymorphic sites were detected by primer extension where the targeted region is amplified; remaining dNTPs are neutralized and

then a terminating extension reaction using a promoter that binds immediately upstream of the polymorphic site as a 'mass modified' nucleotide lacking the 3′-hydroxyl extends the product by a single base [5-9]. The number of CYP2D6 gene copies was detected by qPCR using a 7900HT PCR system [10].

1.14. Data Reporting

Patient reports were generated using myDNA's PGx software (https://www.mydna.life/en-ca/). These reports were uploaded to a secure website accessible to the primary project team by the PI, the User Partner Lead, and the project's Research Assistant. Data was encrypted and only de-identified to the appropriate pharmacist after review by the project team. Genomic reports and patient IDs were sent separately in encrypted Excel spreadsheets. GitHub (https://github.com/) was used to store all analysis routines and to ensure version control.

In addition to genotyping 150 samples, 46 were subjected to Kailos TRS or "target rich sequencing protocol". The NGS data and the final Kailos reports were not returned to the pharmacists and restricted to internal comparisons.

1.15. Patient Consults at the Pharmacy

The reports were released directly to the project leads CN and FADD. At which point they were reviewed before being released to the pharmacist. Reports were reviewed with each participant in a face-to-face appointment with the pharmacist following a standardized script. The pharmacist delivered results, discussed possible therapy change recommendations, and asked if the participant wanted the report shared with the patient's physician. Participants had the option of sharing the report directly themselves or having the pharmacist send a copy. Pharmacists were responsible for recording medication changes. Though all medication changes were made by the patient's physician. All participants were asked to complete a qualitative survey.

1.16. Data Collection and Analysis

To process the myDNA reports for our meta-analysis, each participant's medical considerations and genotypes were extracted from PDFs using tabula [11]. Files were then manually edited to include a patient ID and any potential drug-drug interaction information.

Genotype information from the TRS reports were manually entered into a .csv file and further tidied, such as conversion from wide to long data, using R (version 3.6.1), a programming language for data analysis [12]. To compare genotype calls between TRS and myDNA, only shared alleles were analyzed. A file containing every unique myDNA call was matched with the corresponding Kailos genotype.

Population frequencies for the genotypes CYP2D6, CYP2C19, CYP2C9, and VKORC1 were taken from an analysis of an Australian population [10]. The frequency of CYP2D6 *36 was taken from an American population [13]. The population frequencies of the SLCO1B1, CYP1A2, CYP3A4, CYP3A5, and OPRM1 genotypes were calculated from the global SNP frequency. Global Frequency of the SNPs were gathered from the Genome Aggregation Database (gnomAD) (https://gnomad.broadinstitute.org/) [14]. Hardy-Weinberg equilibrium [15] was used to calculate the genotype frequencies in an ideal population.

All genotype data manipulation and analyses were completed in R version 3.6.1 [Supplement VII]. Analysis depended on R packages: Tidyverse, data.table, reshape2, compare, plyr, and rowr [16-21]. Cost-benefit analysis and tabulation of survey results was completed in Excel. Drug prices were retrieved from the McKesson Canada wholesale drug price list in effect at that time.

1.17. Research Ethics Board Approval & Legal Compliance

Many different pieces of legislation were considered pertaining to personal and health information, pharmacy and pharmacist obligations, and the consent requirements of healthcare interventions. Legal review was obtained to ensure the highest standard of legal compliance.

In developing our Research Ethics Board (REB) procedure, we considered the following Canadian and British Columbian legislation:

1. The Personal Information Protection Act, The Freedom of Information and Protection of Privacy Act, The Health Professions Act and its Bylaws, The Health Care (Consent) and Care Facility (Admission) Act, and The Pharmacy Operations and Drug Scheduling Act. These laws lay out the obligations of the pharmacist, the pharmacy and the University of British Columbia with respect to personal and health information.
2. The Health Professions Act and its Bylaws and The Personal Information Protection Act. These laws governed the pharmacist with respect to the collection, use, disclosure and security of personal and health information.
3. The Freedom of Information and Protection of Privacy Act and the policies of UBC and its Research Ethics Board.

Pharmacists were obligated to follow REB-approved protocols with respect to collection, use, disclosure and security of patient/participant personal and health information under The Health Professions Act and its Bylaws (governing the profession of pharmacists and other healthcare professionals), and the BC College of Pharmacists Code of Ethics and Bylaws (self-governed entity entrusted with protecting public safety by regulating the pharmacy profession).

Researchers were bound by the Protection of Privacy Act (which articulates the access and privacy rights of individuals as they relate to the public sector) and approval from the Clinical Research Ethics Board was obtained. Additionally, an Information Sharing Agreement was initiated by the user partner, governing the relationship and setting out the obligations of the pharmacists and pharmacies with respect to the University. The user partner and their legal counsel acted as an intermediary between the University and the participating corporations in executing each of these agreements. A final piece of legislation that was considered was The Health Care (Consent) and Care Facility (Admission) Act, which regulates the consent requirements for the provision of healthcare services.

1.18. Legal Review

We obtained legal counsel to review the informed consent and data collection protocols developed by the study team prior to deployment. These ethics approved SOPs governed all activities in the study.

References

1. Nunes AP, Oliveira IO, Santos BR, Millech C, Silva LP, González DA, et al. Quality of DNA extracted from saliva samples collected with the Oragene™ DNA self-collection kit. BMC Medical Research Methodology. 2012 May 4;12(1):65.
2. Sullivan GM, Artino AR. Analyzing and interpreting data from likert-type scales. Journal of Graduate Medical Education. 2013 Dec;5(4):541–2.
3. Kailos Genetics. TargetRichTM UMI/IndexAdapters & Sequencing User Manual for PGX [Internet]. Kailos Genetics; 2017 [cited 2020 Jun 4]. Available from: https://d10u8wcbc2zotv.cloudfront.net/media/Kailos_TargetRich_UMI-Sample_Adapter_PGX_Seq_Protocol_August2017.pdf
4. Kailos Genetics. Long-Range PCR for CYP2D6 CNV Analysis. Supplementary procedure for TargetRichTM PGx Assay [Internet]. Kailos Genetics; 2017 [cited 2020 Jun 4]. Available from: https://d10u8wcbc2zotv.cloudfront.net/media/Kailos_2D6CNV_LR-PCR_Protocol2017-RevB.pdf
5. Johansen P, Andersen JD, Børsting C, Morling N. Evaluation of the iPLEX® Sample ID Plus Panel designed for the Sequenom MassARRAY® system. A SNP typing assay developed for human identification and sample tracking based on the SNPforID panel. Forensic Science International: Genetics. 2013 Sep;7(5):482–7.
6. Syrmis MW, Moser RJ, Whiley DM, Vaska V, Coombs GW, Nissen MD, et al. Comparison of a multiplexed MassARRAY system with real-time allele-specific PCR technology for genotyping of methicillin-resistant Staphylococcus aureus. Clinical Microbiology and Infection. 2011 Dec;17(12):1804–10.
7. Bray MS, Boerwinkle E, Doris PA. High-throughput multiplex SNP genotyping with MALDI-TOF mass spectrometry: Practice, problems and promise. Hum Mutat. 2001 Apr;17(4):296–304.
8. Hoffman JM, Dunnenberger HM, Kevin Hicks J, Caudle KE, Whirl Carrillo M, Freimuth RR, et al. Developing knowledge resources to support precision medicine: principles from the Clinical Pharmacogenetics Implementation Consortium (Cpic). J Am Med Inform Assoc. 2016 Jul;23(4):796–801.
9. Ellis JA, Ong B. The massarray® system for targeted snp genotyping. In: White SJ, Cantsilieris S, editors. Genotyping [Internet]. New York, NY: Springer New York; 2017 [cited 2020 Jun 4]. p. 77–94. Available from: http://link.springer.com/10.1007/978-1-4939-6442-0_5
10. Mostafa S, Kirkpatrick CMJ, Byron K, Sheffield L. An analysis of allele, genotype and phenotype frequencies, actionable pharmacogenomic (Pgx) variants and phenoconversion in 5408 Australian patients genotyped for CYP2D6, CYP2C19, CYP2C9 and VKORC1 genes. J Neural Transm. 2019 Jan;126(1):5–18.
11. Aristarán M, Tigas M. Introducing Tabula [Internet]. Source; 2013 [cited 2020 Jun 4]. Available from: https://source.opennews.org/articles/introducing-tabula/
12. R Core Team. R: A language and environment for statistical computing [Internet]. Vienna, Austria: R Foundation for Statistical Computing; 2019 [cited 2020 Jun 4]. Available from: https://www.R-project.org/.
13. Del Tredici AL, Malhotra A, Dedek M, Espin F, Roach D, Zhu G, et al. Frequency of cyp2d6 alleles including structural variants in the united states. Front Pharmacol. 2018 Apr 5;9:305.
14. Karczewski KJ, Francioli LC, Tiao G, Cummings BB, Alföldi J, Wang Q, et al. The mutational constraint spectrum quantified from variation in 141,456 humans [Internet]. Genomics; 2019 Jan [cited 2020 Jun 4]. Available from: http://biorxiv.org/lookup/doi/10.1101/531210
15. Hardy, G.H. Mendelian proportions in a mixed population. Science 1908, 28, 49–50.
16. Wickham H, Averick M, Bryan J, Chang W, McGowan L, François R, et al. Welcome to the tidyverse. JOSS. 2019 Nov 21;4(43):1686.
17. Dowle M, Srinivasan A, Gorecki J, Chirico M, Stetsenko P, Short T, et al. data.table: extension of "data. frame" [Internet]. 2019 [cited 2020 Jun 4]. Available from: https://CRAN.R-project.org/package=data.table

18. Wickham H. Reshaping Data with the reshape Package. Journal of Statistical Software. 2007 Nov 13;21(1):1–20.
19. Murrell P. Compare: comparing objects for differences [Internet]. 2015 [cited 2020 Jun 4]. Available from: https://CRAN.R-project.org/package=compare
20. Wickham H. The split-apply-combine strategy for data analysis. Journal of Statistical Software. 2011 Apr 7;40(1):1–29.
21. Varrichio C. rowr: Row-Based Functions for R Objects [Internet]. 2016 [cited 2020 Jun 4]. Available from: https://cran.r-project.org/web/packages/rowr/index.html
