# Supplementary material for: Pharmacogenomics at the Point of Care: A Community Pharmacy Project in British Columbia
Source: J Pers Med. 2020 Dec 24;11(1):11. doi: 10.3390/jpm11010011 (PMC7823931; doi:10.3390/jpm11010011)
Supplement: Supplementary file 1 [file jpm-11-00011-s001.zip › Community_PGx_Supplement/Supplement_I-Recruitment_Checklist.pdf]

a place of mind
THE UNIVERSITY OF BRITISH COLUMBIA

PARTICIPANT RECRUITMENT CHECKLIST
Pharmacogenomic Services in Community Pharmacy
(H16-02362)

Date		
**Ensure you complete and document the following steps** when recruiting a participant for the study so they may provide consent. **Write down any relevant notes/discussion that come up during the process.**		
☐	Move participant into private area – verify they have about 20-25 mins.	
☐	UBC Pharmaceutical Sciences leading the research, funded by Genome BC, Pfizer and Green Shield.	
☐	Participation is voluntary, won't have any effect on healthcare they receive from pharmacy or other healthcare providers whether they participate or not.	
☐	Project is looking to recruit participants who:<br>• are currently taking or have previously taken one of the qualifying drugs<br>• understand English or have a competent translator<br>• are 19 years or older<br>• are able to provide consent & info required	
☐	You and the research team have been trained on consent procedures, handling saliva samples and maintaining privacy & confidentiality in compliance with applicable laws.	
☐	Identify the purposes of the research project:<br>1. Assess public and patient receptivity to receiving pharmacogenomic sequencing and advice in community pharmacies.	
☐	2. Outcome analysis of the test results.	
☐	3. Validate storage and communication of actionable data	
☐	4. Capture patient experiences.	
	5. Creating digital database of DNA sequence info for future research.	
	6. Inform the heath care case and financial case for PGx implementation.	
☐	Participation advances genomic research. Participant will receive test results and medication may be altered.	
☐	Clearly explain what you require of the participant and re-iterate what the study requires:<br>• Consent	
☐	• Saliva sample	
☐	• Personal info (may contact if something missing or for future research)	
☐	• Medication/medical history – will be linked to DNA info for research purposes	
☐	Explain/reassure the participant about what will happen to their personal info:<br>• Protected by privacy laws at all times.	
☐	• Only project manager, principle investigator or his designate will have access to identifying info.	
☐	• Once participant info & saliva sample received at UBC, will only be identified by participant code.	

☐	• Key file will be only link between participant codes & identifying info. The key file, will be transferred to a separate drive which will be kept in a secure location within UBC with limited access.	
☐	• No personal info sold or used for commercial purposes. Identity will not be disclosed in any report.	
☐	• All identifying info and saliva samples will be destroyed by December 31, 2020. De-identified digital DNA sequences retained until no longer useful for research.	
☐	Re-iterate: A project goal is to create a digital database of DNA sequence info – means that de-identified DNA info could be used for other research (with ethics approval).	
☐	Can withdraw any time without giving reason (until key filed destroyed - Dec 31, 2020).	
☐	Can contact you or contacts listed in info package for questions or withdrawal.	
☐	By consenting, participant does not give up any legal rights if they become ill or injured as a result of participation.<br>• Risk is extremely low since – only providing saliva sample & some personal/medical info.	
☐	Study will not cost anything except their time. Will receive a report for participation.	
☐	Show video to explain some science and procedures. Stay with them to answer questions.	
☐	Ask participant to explain what they understand and think of the info they've heard and seen. Any questions or more information required?	
☐	Explain what the participant needs to do next:<br>1. Take Participant Info Package and Consent Form home to review.	
☐	2. Understand their obligations and how their info, saliva and DNA is going to be used.	
☐	3. If they still want to participate, a second meeting is required to:<br>a. Sign the consent form.<br>b. Collect the saliva sample.<br>c. Obtain some personal and medical info.<br>i. Show Data Collection Form & Participant Identifying Form	
☐	Arrange follow-up meeting.<br>• Bring in meds or list of meds.<br>• Must not eat, drink, smoke, or chew gum 30 mins prior to second meeting for saliva collection.	

Pharmacy Name			
Pharmacy Address			
Pharmacist Name		License #	
Pharmacist Signature			
