# Supplementary material for: Pharmacogenomics at the Point of Care: A Community Pharmacy Project in British Columbia
Source: J Pers Med. 2020 Dec 24;11(1):11. doi: 10.3390/jpm11010011 (PMC7823931; doi:10.3390/jpm11010011)
Supplement: Supplementary file 1 [file jpm-11-00011-s001.zip › Community_PGx_Supplement/Supplement_II-Information_&_Consent.pdf]

a place of mind
THE UNIVERSITY OF BRITISH COLUMBIA

AFFIX BARCODE LABEL HERE
(1D Alphanumeric Barcode)
Do not photocopy once barcoded

PARTICIPANT INFORMATION and CONSENT FORM

Pharmacogenomic Services in Community Pharmacy
(H16-02362)

Principal Investigator: Dr. Corey Nislow, Ph.D.
Faculty of Pharmaceutical Sciences
The University of British Columbia (UBC)
604-827-1579

Co-Investigator: Derek Desrosiers, BSc(Pharm), RPh
British Columbia Pharmacy Association
Vancouver, BC
604-269-2862

Sponsors:
British Columbia Pharmacy Association
Green Shield Canada
Pfizer Canada
Genome BC
The University of British Columbia – Faculty of Pharmaceutical Sciences

Study Contact Numbers: Dr. Corey Nislow, 604-827-1579
Mr. Derek Desrosiers, 604-269-2862

1. INVITATION TO PARTICIPATE IN A RESEARCH PROJECT

You are being invited to participate in this study because you are undergoing, or have previously undergone, drug therapy with at least one of the drugs listed in the appendix to this document and are familiar with your pharmacist, who has completed research ethics training and enrolled in this study.

The study aims to develop methods to optimize medication efficacy, reduce adverse effects and improve drug treatment outcomes using the individual's genetic information (DNA). Your pharmacist is participating in this study under the BC College of Pharmacists Code of Ethics, Standard 5: Registrants Participate in Ethically Valid Research.

2. YOUR PARTICIPATION IS VOLUNTARY

Your participation is entirely voluntary. You have the right to refuse to participate in the study. Before you decide, it is important for you to understand what the study involves. This consent form will tell you about this study, the purpose of the study, your role in the study, and the possible benefits, risks and discomforts. You also need to know that there are important differences between being in a research study and being cared for by your pharmacist or physician. The goal of the study is to help the researchers obtain new knowledge so as to improve health outcomes from your drug therapy and help patients in the future with their drug therapy. At the same time, the researchers and your pharmacist have a duty of care to all participants and will inform you of any information that may affect your willingness to remain in the study.

If you wish to participate, you will be asked to sign this form. If you decide to take part in this study, you are still free to withdraw at any time without giving any reasons for your decision. Your medical care will not be negatively affected if you choose to withdraw from the study. This consent form describes the procedures (Section 11) that are being carried out for research purposes. Please review the consent document carefully when deciding whether or not you wish to be part of the research and sign this consent only if you accept being a research participant. Everything we plan to do in this research study with your personal information (identifying, demographic and medical information detailed in Section 8), saliva sample, DNA sample extracted from the saliva and your DNA sequence is described in this consent form.

Please take time to read the information provided here carefully and ask questions to the pharmacist who will provide you additional information you need. You will also be provided a video that describes genomics. Please ask for advice if you think you need it.

3. WHO IS CONDUCTING THIS STUDY?

This study is conducted by researchers in the Faculty of Pharmaceutical Sciences at the University of British Columbia and the BC Pharmacy Association. Funding for the study has been provided by Green Shield Canada, Pfizer Canada and Genome BC. For further clarity, Green Shield Canada and Pfizer Canada have no role in conducting the study and will not receive any study information other than that which is publicly published after the completion of the study.

4. BRIEF BACKGROUND

Because a person's DNA sequence is unique to them and does not change in the vast majority of the cells of your body, your DNA sequence can, with your permission, be repeatedly queried (in a manner similar to a database search) for any drug you may be prescribed currently or in the future.
Among other uses, in a few instances, it can help determine disease diagnosis and prognosis, and appropriate medication therapy. Very little public awareness exists about the potential value of genomic information, and, in particular, the potential values of using genome data to more effectively manage medication therapy – known as 'pharmacogenomic testing'.

A practical application of genome science, pharmacogenomic testing can help predict the efficacy of medications for individuals. In such testing, individual genomic data is gathered to determine the appropriateness of a particular medication for a patient, and need not focus on disease diagnosis or prognosis. Because medications can be targeted to those who will likely benefit, and avoided by those who will likely not, this, in a few instances, has the potential to improve individual health care outcomes and help governments and other payers manage escalating health care costs. By ensuring patients receive only those medications and dosages known to be effective for them, provincial governments and private insurers, in the future, can avoid the costs of using expensive, yet ultimately ineffective, medications.
The science of how genetic interactions with drug therapy has developed over the past forty years. Like all clinical information, there is a spectrum of evidence for how useful a particular intervention is for a particular patient. For this study, we are using information with a very high level of evidence. Specifically, for the gene variants we require one of the following criteria to be met 1) A DNA sequence that has been reviewed by medical experts to be relevant to certain drugs and 2) only variants listed by the United States Food and Drug Administration will be tested. https://www.fda.gov/drugs/scienceresearch/researchareas/pharmacogenetics/ucm083378.htm.

The science and application of genetic testing is a rapidly developing field. Because drug recommendation and specific doses are designed to serve the largest population possible, we can predict that every individual participant will identify at least one variant that is relevant for at least one medication they are currently taking or will take in the future. That said, as with any clinical assessment, it is not clear at this time if the benefits of making a drug or dosage change outweigh the risks.

5. WHAT IS THE PURPOSE OF THE STUDY?

The Pharmacogenomic Services in Community Pharmacy project is working with pharmacists at 20-30 community pharmacies throughout BC to recruit up to 200 patients to demonstrate that genomic data can be collected via saliva sample by community pharmacists and to use that data to generate clinically actionable reports for your pharmacist and physician to use to make more informed decision about your drug therapy. The proposed use of community pharmacies as the point of delivery for this service is unique in Canada. The project aims to demonstrate that delivery of pharmacogenomic services can be done in pharmacy effectively and without compromising patient privacy.

This study will utilize Standard Operating Procedures (SOPs) developed in a previous study for, amongst others, sample collection and transportation to the UBC Sequencing Centre for processing and sequencing. We will also develop a training program for community pharmacists, along with educational materials for patients. The end-product will be analysis of genetic information, generation of a clinically actionable report, validation of the storage and communication of the data. A key component is to ensure you understand both the nature of the data collection process, as well as how this information can – and cannot – be used to address issues of your personal health.

The study will also use genetic information obtained from sequencing your DNA to retrospectively analyze your drug therapy and provide recommendations for possible changes in therapy to optimize effectiveness, and reduce adverse effects. As part of ongoing research by the investigators, your DNA sequence is being used to create a digital database so we can learn more about how differences in our DNA can affect our health and wellness. In other words, the project also aims to develop a registry of DNA sequence information to allow for further research. This is why your DNA sequence will not be destroyed upon completion of the study.

6. WHO CAN PARTICIPATE IN THE STUDY?

You may be able to participate in this study if you:

- Are undergoing drug therapy with at least one of the drugs listed in the appendix to this document
- Understand English or have a competent person translate for you
- Are 19 years or older
- Able to provide the information described in section 8
- Able to consent using the form provided in this package

7. WHO SHOULD NOT PARTICIPATE IN THE STUDY?

There are no criteria that would exclude you from participating as long as you have been identified by a participating pharmacist as meeting the criteria for the study. If you are willing to provide your saliva sample and identifying, demographic and medical information and meet the criteria above, then you are able to participate.

8. WHAT ARE MY RESPONSIBILITIES?

If you decide to take part in this study, the pharmacist will collect a saliva sample from you as well as the information listed below.

- Identifying Information
    - Name (as it appears on Care Card)
    - Personal Health Number
    - Address (optional)
    - Telephone Number (optional)
    - E-mail (optional)
- Demographic Information
    - Month and year of birth

  - Gender
  - Height (optional)
  - Weight (optional)
  - Ethnic Background (optional)
- Medical Information
  - Current medications including dosage and directions for use
  - Over the counter (OTC) drugs if used
  - Allergies
  - Medical conditions
  - Identified adverse drug events
  - Laboratory test results (optional)

You will also be asked to complete a post-study experiential survey and you may also be asked to participate in a voluntary post-study interview conducted by one of the investigators.

9. WHAT ARE THE POTENTIAL BENEFITS OF PARTICIPATING?

Benefits to You

The direct benefit to you will be a clinically actionable report that may assist your pharmacist and/or physician in making possible adjustments to your drug therapy to ensure you're a getting the maximum benefit from the drug(s) while minimizing adverse effects. Your DNA sequence is being used to develop a pharmacogenomics database that will contribute to other patients receiving tailored drug therapy based on their DNA sequence.

Benefits to Others

We hope that the information learned from this study can be used in the future to benefit people in British Columbia.

10. WHAT ARE THE POSSIBLE HARMS AND DISCOMFORTS?

Physical Risks

The only physical action you will be asked to do is provide a saliva sample. This does not involve serious problems for most people.

Social or Emotional Risks

Your insurer or employer may at some time in the future attempt to request access to your genetic information from the DNA sequence stored at UBC. Since the collection and sequencing of this information has been done for the limited purpose of research, the data cannot be disclosed for such requests. UBC will prohibit such disclosures, as maintaining your privacy is one of our main responsibilities.

11. STUDY PROCEDURES

If You Decide to Join This Study: Specific Procedures

If you agree to take part, the following procedures, which are expected to take approximately 25 minutes of your time, will occur:

**Specific Procedures** (all overseen by a BC pharmacist registered in our study)		
**Saliva Sample Collection**	**Collect Personal Information (see 8 above)**	**Additional Clinical Information**
**Frequency?** Once only<br>**How much?** 1 tube (2mL/tube)	Identifying Information<br>Demographic Information<br>Medical Information	If you consent, you may be contacted and invited to participate in additional research at a later date to provide additional or clarifying the Medical Information

We will:

- Take a total of 1 mL of saliva sample (about ½ teaspoon) by asking you to spit into a tube. We will do this one time only and it will take place in the pharmacy.
- Collect your identifying and demographic information as listed in section 8, as provided by you on the Data Collection Form.
- Collect your medical information as listed in section 8, as provided by you on the Data Collection Form.
- Send your saliva sample to the lab at UBC

The UBC Sequencing Centre lab in the Faculty of Pharmaceutical Sciences at UBC will:

- Extract your DNA sample from your saliva sample.
- Separate the approximately 2% portion of your DNA that contains nearly all 20,000 genes and “read” it to identify anomalies and generate your actual personal DNA sequence.
- Perform gene panel sequencing of up to 340 fragments of your DNA to focus on variants that are known to alter the response to medications
- Securely store your DNA sequence at the UBC Sequencing Centre for as long as it remains useful for research purposes for this study or by other researchers.
- Send your de-identified DNA sequence data to myDNA Australia in Melbourne Australia so that they can generate a clinically actionable report that will be sent to your pharmacist and physician (if you agree) to be reviewed with you, potentially resulting in modifications to your drug therapy.

The review of your report with your pharmacist is expected to take an additional 20-25 minutes.

12. WHAT ARE THE ALTERNATIVES TO PARTICIPATION IN THIS PART OF THE STUDY?
The study is not related a new remedy or medication for your healthcare. As such, not participating in this study will have no adverse effect on the health care you receive from your pharmacist, physician(s), or other health care professional.

13. WHAT HAPPENS IF I DECIDE TO WITHDRAW MY CONSENT TO PARTICIPATE?
You can stop participating in the study at any time without giving reasons. If you withdraw your consent, the medical care you receive from your pharmacists, physician(s), or other health care

professional will not change. You do not have to give any explanation for why you wish to withdraw.

There are two types of withdrawals you may choose from:

1. **Withdraw consent for any future activities of this study but allow continued access to information.** This means that you will no longer be contacted or asked to participate in ongoing study activities. The project will continue using the information you already provided and continue to access information in the study database.
2. **Withdraw consent for past and future activities of the study.** This means that you can no longer be contacted and we can no longer access any information about you. All the data and samples already collected about you will be destroyed.

Depending on the type of withdrawal you choose the withdrawal process and deletion of your information, if required, will also be implemented at the pharmacy at which you enrolled.

If you wish to stop participating at any time, please contact the Principal Investigator, Dr. Corey Nislow: Telephone: 604-827-1579
E-mail: corey.nislow@ubc.ca
Mail: Dr. Corey Nislow, 6619-2405 Wesbrook Mall, Vancouver BC, V6T 1Z3

14. CAN I BE ASKED TO LEAVE THE STUDY?
The study investigators may decide to ask you to leave the study if they feel that it is in your best interests. You may be withdrawn from the study, for example, if you no longer meet the study requirements. Again, the medical care you receive from your pharmacist and other health care professionals remains unchanged regardless of whether or not you are a participant in the study. At that time of withdrawal, the Principal Investigator will contact you and explain the steps being undertaken.

15. HOW WILL MY PERSONAL & HEALTH INFORMATION BE PROTECTED?
Applicable Laws: At all times, your personal and health information is protected by *The Personal Information Protection Act, The Freedom of Information and Protection of Privacy Act*, *The Health Professions Act* and its *Bylaws*, *The Health Care(Consent) and Care Facility (Admission) Act,* and *The Pharmacy Operations and Drug Scheduling Act.* These laws lay out the obligations of the pharmacist, the pharmacy and the University of British Columbia with respect to personal and health information.

The pharmacist obtaining your consent is subject to the obligations with respect to the collection, use, disclosure and security of your personal and health information under *The Health Professions Act* and its *Bylaws* and *The Personal Information Protection Act.* They are also trained by the UBC research team in the process of obtaining consent and the confidentiality obligations in the conduct of research study.

The researchers are UBC employees and will use and retain personal and health information under *The Freedom of Information and Protection of Privacy Act* and the policies of UBC and its Research Ethics Board. Your confidentiality will be respected. However, research records or other source records identifying you may be inspected in the presence of the Investigator or his designate by representatives of the British Columbia Pharmacy Association and/or Genome BC and UBC Clinical Research Ethics Board for

the purpose of monitoring the research.

The other sponsors of the study will never have access to your information. UBC may disclose your DNA sequence to other researchers who receive necessary ethical and legal approval.

Coding: You will be assigned a unique Participant Code as a participant in this study (for example, 'Participant ABC-001'). This code will be used instead of your identifying information on all study-related information collected from you, so that your identity as a participant in this study will be kept confidential. Information that identifies you will be known only to the Principal Investigator, his designate and your pharmacist who collected the information at the outset.

The list that matches your name to the unique code that is used on your research-related information (i.e. the 'coding list') will be securely stored and access limited to three individuals at UBC. None of your personal information (section 8) will be sold to anybody or used for commercial purposes and your identity will not be disclosed in any report.

Data Management and Security: Once collected from you after your consent, your personal information (identifying) will be stored in a secure form at the participating pharmacy and transferred securely to the study researchers at UBC Pharmaceutical Sciences. At UBC, storage of this information is subject to the UBC Information Security Standards under Policy #104, Acceptable Use and Security of UBC Electronic Information and Systems. Personal identifying information will be removed and only the Participant Code will be used thereafter. The consent form and your identifying information with the link to your Participant Code will be stored in an encrypted form in a separate password protected file on a different server at UBC. The key file, after coding described above, will be transferred to a separate hard drive which will be kept in a safe within UBC with limited access.

Research using your coded information might someday lead to the development and sale of a medical or genetic test or product. This may be done by a university of hospital, or a commercial company, because sometimes researchers are funded by or work with companies. This means that in the future researchers, including potentially commercial companies, may benefit financially. However, you will not gain any personal financial advantage from this commercialization.

Retention period and sharing of your information: The long-range goal of this study is to develop a DNA sequence database (the registry described in Section 5) that may be used to predict the potential and level of efficacy or degree of adverse events that may occur in an individual depending upon the drug prescribed for a particular disease therapy and the individual's personal gene sequence.

Other researchers might request from the principal investigator the use of your DNA sequence for other projects in the future. The DNA sequence does not link to your identifying, demographic or medical information. Any such access will be subject to the ethical and legal approval.

Destroying your personal information: All of your personal information (identifying, demographic and medical information), including your saliva and DNA sample, will be destroyed no later than December 31, 2020.

Any study related data and/or samples, sent outside of Canadian borders may increase the risk of disclosure of information because the laws in those countries, (for e.g. the Patriot Act in the United States) dealing with protection of information may not be as strict as in Canada. However,

all study related data and/or samples, that might be transferred outside of Canada will be coded (this means it will not contain your name or personal identifying information) before leaving the study site. By signing this consent form, you are consenting to the transfer of your information and/or samples, to organizations located outside of Canada.

- myDNA Australia

If you have any questions with regards to your collection, use, management or security of your personal information or any other issue before completing the consent process, please ask the pharmacist to clarify. At any later time, should you have more questions or need clarifications please use the contacts in Section 18 and 19.

16. WHAT HAPPENS IF SOMETHING GOES WRONG?

By signing this form, you do not give up any of your legal rights and you do not release the study doctor, participating institutions, or anyone else from their legal and professional duties. If you become ill or physically injured as a result of participation in this study, medical treatment will be provided at no additional cost to you. The costs of your medical treatment will be paid by your provincial medical plan.

17. IS THERE ANY FINANCIAL GAIN OR COST TO ME TO PARTICIPATE?

Costs

This study will not cost you any money, other than the time it takes for you to participate in the study procedures at the pharmacy collecting your personal information, medication history and saliva sample after obtaining your consent.

18. WHO DO I CONTACT IF I HAVE QUESTIONS ABOUT THE STUDY DURING MY PARTICIPATION?

If you have any questions or desire further information about this part of the study before or during participation, you can contact:

Co-Investigator: **Mr. Derek Desrosiers, 604-269-2862**

Principal Investigator: **Dr. Corey Nislow, 604-827-1579**

19. WHO DO I CONTACT IF I HAVE ANY QUESTIONS OR CONCERNS ABOUT MY RIGHTS AND/OR EXPERIENCES AS A SUBJECT DURING THE STUDY?

If you have any concerns about your rights as a research participant and/or your experiences while participating in this study, contact the Research Participant Complaint Line in the University of British Columbia, Office of Research Ethics at by e-mail at RSIL@ors.ubc.ca or by phone at 604-822-8598 (Toll Free: 1-877-822-8598).

.

20. AFTER THE STUDY IS FINISHED

The DNA sequence will be securely stored digitally at UBC for as long as it remains useful for research purposes for this study or by other researchers – this could be for decades. You will not be given the DNA sequence because these results will not benefit you or your family. You will,

however, be given the results of your genetic tests in the form of a clinically actionable report. The general study results using coded data provided by all participants will be published in articles, poster presentations, or talks. These results are expected to be publically available in a few years' time. When your DNA sequence is sent to myDNA Australia to generate your report, it will be linked to your demographic and medical information but not your name.

APPENDIX

Antidepressants	Antipsychotics
Agomelatine	Aripiprazole
Amitriptyline	Clozapine
Citalopram	Haloperidol
Clomipramine	Olanzapine
Dothiepin	Quetiapine
Duloxetine	Risperidone
Escitalopram	Zuclopenthixol
Fluoxetine	
Fluvoxamine	
Imipramine	
Mianserin	
Mirtazapine	
Moclobemide	
Nortriptyline	
Paroxetine	
Sertraline	
Trimipramine	
Venlafaxine	
Vortioxetine	

a place of mind
THE UNIVERSITY OF BRITISH COLUMBIA

AFFIX BARCODE LABEL HERE
(1D Alphanumeric Barcode)

Do not photocopy once barcoded

Pharmacogenomic Services in Community Pharmacy
Principal Investigator: Dr. Corey Nislow, Associate Professor, UBC

Principal Investigator: **Dr. Corey Nislow, 604-827-1579**

Co-Investigator: **Mr. Derek Desrosiers, 604-269-2862**

Participant Consent Form

My pharmacist explained the research project objectives, the benefits and potential harms and discomforts to me and answered the questions posed by me. In addition, I have read and understood the information provided to me by my pharmacist, in writing and presented in a video. I have been able to reflect on the request for information and ask for advice if needed.

I understand that:

- my pharmacist will collect my saliva, identifying information, demographic information and medical information
- the information collected will be kept confidential and secure as described in the participant information section
- my participation is completely voluntary and I can withdraw from the study at any time
- this study may not provide me any direct benefits for participation
- I will receive a copy of this signed consent form for my records
- I may contact the pharmacist or study team at any time to access or correct my personal information
- I will not be provided my DNA sequence however I will be provided genetic test results in a clinically actionable report
- I am not waiving any of my legal rights as a result of signing this consent form

I consent to:

- participate in this study
- the pharmacist identified below collecting my identifying, demographic and medical information from me and sending it to University of British Columbia for use in the research study “Pharmacogenomic Services in Community Pharmacy”
- the pharmacist, on behalf of the study team, taking my saliva sample for use in the study as described in the participant information section
- my saliva sample with my identifying, demographic and medical information to be sent to the University of British Columbia for use in the research study “Pharmacogenomic Services in Community Pharmacy” as described in the participant information section
- my genetic data being sent securely, along with my demographic and medical information in a de-identified manner to myDNA Australia to have a clinically actionable report generated
- the pharmacist reviewing the results of my genetic tests (clinically actionable report) with me

- my DNA sequence information to be securely stored at UBC for as long as it remains useful for research purposes for this study or by other researchers
- my DNA sequence information to be shared with other research groups who have obtained necessary ethical and legal approval
- being contacted to participate in additional research to providing additional medical information or clarifying information provided

**Participant Full Name**	**Signature**	**Date Signed**

**Full Name of person assisting participant with consent (if present)**	**Signature**	**Date Signed**

**Full Name and License Number of Pharmacist obtaining consent**	**Signature**	**Date Signed**
