# Supplementary material for: Pharmacogenomics at the Point of Care: A Community Pharmacy Project in British Columbia
Source: J Pers Med. 2020 Dec 24;11(1):11. doi: 10.3390/jpm11010011 (PMC7823931; doi:10.3390/jpm11010011)
Supplement: Supplementary file 1 [file jpm-11-00011-s001.zip › Community_PGx_Supplement/Supplement_IV-Video_Script.pdf]

Pharmacogenomic Services in Community Pharmacy
(H16-02362)

Informational Video Script

First Half – Genomics 101

Our bodies, like all living things, are made up of cells. Inside each cell is something called DNA, which is a molecule that determines whether we're a tree, or a dog, or in our case, a human. DNA, or deoxyribonucleic acid, contains four main molecules, called bases, represented by the letters A, C, T or G. These molecules pair up and string together to form a winding ladder 3 billion bases long, called a **genome**.

If you were compare the DNA of one human to another, of those 3 billion letters, 99.9% are the same. It is then that 0.1% difference that makes each of us unique. The differences in each individuals genome that determine who we are is referred to as our **genotype**.

We all can see that we're different on the outside, but the differences don't stop there. Our genome also can determine how our body responds to different medications. All medications have the potential to cause side effects. These can range from mildly annoying, like a rash or an upset stomach, to severe or life threatening. Little is known about how or why these side effects occur, and it is frustratingly difficult to predict them.

Part of the answer to the puzzle lay in our genes. **Genes** are specialized segments of DNA that provide the instructions for how our bodies develop and function. While every individual has a similar set of 20,000 genes, some have certain gene variants "or flavors" that increase the chances of experiencing certain side effects. In the same way that a number of gene variants combine to specify how tall you are, different gene variants combine to predispose you to side effects.

The results of these gene combinations are called traits, or more technically, **phenotypes**. It is essential to appreciate that phenotype is not completely predetermined by our genetics – Genes are half of the story; the environment is the other half. When we say environment, this means everything besides our genes, the meal you ate in the morning is part of your environment, just as is the drug you took with your meal.

Because environment can vary so dramatically, it is essential to consider both sides of the equation when thinking about drug effects. This is the essence of what we call **pharmacogenomics**.

Second Half – Study 101

Fortunately, we can perform genetic tests to find out what types of genes you have. Until very recently, we tested genes one at a time, at a cost of several hundred dollars per test. Recent advances in technology now allow us to sequence or “read” the DNA of all 20,000 genes in a single test starting from a simple saliva sample. We then compare readings between different people to find matches between gene variants and side effects.

The purpose of our study is to learn more about which gene variants are associated with different responses, or side effects, to medication. You have been provided with a detailed consent form you can follow along with which will expand upon what this study involves.

We are doing these genetic tests on participants who receive care at one of the listed study sites. While you may be able to participate in the study if you meet the listed criteria, we also have to make sure there are no reasons why you shouldn’t participate in this study. If you decide to join this study, there will be specific procedures carried out related to collecting your saliva sample, and you will be put into a certain study group. The saliva samples we collect from our participants, and the data we get from them, will be stored in a ‘biobank’ for many years, which will allow us to perform more studies on your DNA in the future.

By using these methods, we hope to improve our knowledge of how medications work. This type of research will help doctors provide the most effective form of treatment to the people being treated by the medications we’re studying. Keep in mind, your privacy is of the utmost importance to us, and we will take all measures to maintain your confidentiality. This is provided in your consent form.

Hopefully this provides you with an understanding of what genetics is, and you would like to help with our research. Please read carefully and make sure you understand the consent form, and ask your pharmacist or our study contact listed any questions you may have.

We thank you for your time and hope you join our research about how to make medications safer and more effective.

At the conclusion of the video, before asking the participant if they have any questions, the pharmacist will read the following statement:

Based on your report, the pharmacist, will do one of the following

(i) Take no action and send a copy of the report to your physician to add to your medical record.
(ii) Make a change to your drug therapy by either changing a dose, discontinuing a medication or making a therapeutic substitution to change a medication. I will initiate

the change and within 24 hours report the details of the change, in writing, to your physician along with a copy of the report.

(iii) Recommend to your physician to either change a dose, discontinue a medication or make a therapeutic substitution to change a medication.
