# Supplementary material for: Pharmacogenomics at the Point of Care: A Community Pharmacy Project in British Columbia
Source: J Pers Med. 2020 Dec 24;11(1):11. doi: 10.3390/jpm11010011 (PMC7823931; doi:10.3390/jpm11010011)
Supplement: Supplementary file 1 [file jpm-11-00011-s001.zip › Community_PGx_Supplement/Supplement_IX-Pharmacy_Locations.pdf]

Pharmacy	City
Armstrong Pharmacy	Armstrong
BC Pharmacy Association	Kamloops
Harbourside Pharmachoice	Port McNeill
Hart Drug Mart	Prince George
Heart Pharmacy Ida	Victoria
Lakeside Medicine Centre	Kelowna
London Drugs #67	Courtenay
Naz's Pharmacy No. 2 Ltd.	Surrey
Pharmasave #032	Houston
Phoenix Dispensary	Prince George
Pratt's Compounding Pharmacy	Kamloops
Quadra Village Drug Mart	Victoria
Safeway #4918 (Willowbrook)	Langley
Save-on-foods Pharmacy #987	Williams Lake
Shoppers Drug Mart #2212	Surrey
Wellness Pharmacy #1	Vancouver
Wilson Pharmacy	Port Coquitlam
