# Supplementary material for: Pharmacogenomics at the Point of Care: A Community Pharmacy Project in British Columbia
Source: J Pers Med. 2020 Dec 24;11(1):11. doi: 10.3390/jpm11010011 (PMC7823931; doi:10.3390/jpm11010011)
Supplement: Supplementary file 1 [file jpm-11-00011-s001.zip › Community_PGx_Supplement/Supplement_V-Pharmacist_Survey.pdf]

a place of mind
THE UNIVERSITY OF BRITISH COLUMBIA

British Columbia
Pharmacy Association

PHARMACIST EXPERIENCE SURVEY
Pharmacogenomic Services in Community Pharmacy
(H16-02362)

Please indicate how much you agree or disagree with the following statements:

	Strongly Disagree	Disagree	Agree	Strongly Agree
1. The training for participation in this study was adequate and appropriate.				
2. The support from the study team met my needs.				
3. The study team responded to my queries in a timely manner.				
4. The materials I received for the study were thorough.				
5. I feel my patients appreciated the opportunity to participate in this study.				
6. Overall, I had a positive experience participating in this study.				

Additional Comments:
