# Supplementary material for: Pharmacogenomics at the Point of Care: A Community Pharmacy Project in British Columbia
Source: J Pers Med. 2020 Dec 24;11(1):11. doi: 10.3390/jpm11010011 (PMC7823931; doi:10.3390/jpm11010011)
Supplement: Supplementary file 1 [file jpm-11-00011-s001.zip › Community_PGx_Supplement/Supplement_VI-Patient_Survey.pdf]

a place of mind
THE UNIVERSITY OF BRITISH COLUMBIA

British Columbia
Pharmacy Association

PARTICIPANT EXPERIENCE SURVEY
Pharmacogenomic Services in Community Pharmacy
(H16-02362)

Please indicate how much you agree or disagree with the following statements:

	Strongly Disagree	Disagree	Agree	Strongly Agree
1. My pharmacist gave me enough information about the study.				
2. I understood the purpose of the study and the terms used by my pharmacist were not overly technical.				
3. I was given enough time to ask questions and discuss the study with my pharmacist.				
4. I feel comfortable participating in this study.				
5. I think the community pharmacy is a good location for genetic testing.				
6. I am comfortable with the idea of pharmacists using genetic information to help them make drug therapy decisions.				
7. I would be willing to participate in this type of research again.				

Additional Comments:
