# Supplementary material for: Pharmacogenomics at the Point of Care: A Community Pharmacy Project in British Columbia
Source: J Pers Med. 2020 Dec 24;11(1):11. doi: 10.3390/jpm11010011 (PMC7823931; doi:10.3390/jpm11010011)
Supplement: Supplementary file 1 [file Supplement_VIII-Example_Report.pdf › Community_PGx_Supplement/Supplement_VIII-Example_Report.pdf]

6 December 2018

Dr Corey Nislow
University Of British Columbia Sequencing And Bioinformatics Consortium
Pharmaceutical Sciences Building Room 3124
Vancouver, BRITISH COLUMBIA, V6T 1Z3

Dear Dr Nislow,

Please find attached your patient's myDNA report.

Patient Full Name: Patient S81-252
Patient Address: 6619-2405 Wesbrook Mall, Vancouver, BC, V6T 1Z3
DOB: 1-Mar-1965
Reference ID: 81995N4T3V1
Patient Phone Number: +16048271579

This myDNA medication pharmacogenomic test was ordered by your patient and they nominated you to receive a copy of their results.

The myDNA medication test is a pharmacogenomic test which looks at common genetic variants in a number of genes with likely clinical significance and potential to enhance safe and effective prescribing of a range of medications. The information provided by the test is mainly around drug metabolism and how genotype-predicted changes influence plasma concentrations, and clinical effects (both therapeutic and adverse). The reports prepared by the myDNA clinical team, provide suggestions on medication selection, dose modification and other clinically relevant information. This information is based on the published literature, as well as peer-reviewed pharmacogenomic guidelines where available.

This report is not sent directly to the patient. The results and report are delivered to the patient by a registered healthcare professional, who is either 1) a requesting doctor, 2) a requesting community pharmacist or 3) the patient’s self-nominated doctor if the test was conducted with a self-administered kit. Following the delivery of the results by the relevant healthcare professional, the patient is given access to a secure online portal at www.mydna.life/explore where they can view their report and receive simple explanations of the results.

As pharmacogenomics is a relatively new area of medicine which clinicians are incorporating into their practice, our service believes it is vital to provide timely support. Therefore, we have a clinical team available by phone to answer any questions you may have about this report, including interpretation and clinical utility, or about pharmacogenomics in general. The clinical team can be reached on 1-844-472-7896.

Kind Regards,

A/Prof Les Sheffield
Medical Director
MyDNA Life

ABOUT THIS REPORT

Overview

This report provides clinically relevant information on what the patient's genetic results predict about their response to a number of medications covered by this report.

The information concerns drug metabolism and plasma concentrations (drug exposure), as well as the potential for altered clinical effects.

Based on the available information found in the published literature, each medication has been assigned a category according to the likely clinical significance of each gene-drug interaction.

The three categories are:

- Major – significant result that may require altering this medication
- Minor – result should be considered as may affect medication response
- Usual – usual prescribing considerations apply

For many medications covered in this report, international, peer reviewed prescribing guidelines are available and these are included in our report.

The two major guidelines are those of the Clinical Pharmacogenetics Implementation Consortium (CPIC) and the Royal Dutch Pharmacists Association – Pharmacogenetics Working Group (DPWG).

Report breakdown

The report consists of the following sections:

- Report Summary – identifies which of the patient's listed medications have pharmacogenomic information relevant to the genes tested, with an indication of the clinical importance of this information (i.e. "Major", "Minor" or "Usual" prescribing considerations).
- Genetic Test Results Overview – genotype result for the eight gene test (i.e. six genes encoding CYP450 metabolising enzymes relevant to a large number of medications, *VKORC1* which relates to warfarin sensitivity and *SLCO1B1* which relates to statin induced myopathy).
- Current Medications – details of the interaction between the patient's genetic results and their medication, based on the current scientific literature, as well as clinical recommendations, many sourced from peer-reviewed, published guidelines.
- Potential Drug Interactions – identifies which of the patient's listed medications can significantly inhibit or induce CYP enzymes, as they may modify the genotype-predicted enzyme function.
- Future Medications – lists medications that the patient is not currently taking that have potentially clinically significant prescribing considerations based on the patient's genetic test results (also classified as having "Major", "Minor" or "Usual" prescribing considerations).

As part of our clinical service, we have a team of clinical experts available to answer any questions you may have about this report or about pharmacogenomics in general.

If you have any such queries, please call our clinical team on 1-844-472-7896.

myDNA Medication

Personalised Medication Report
for *Patient S81-252*

Name:	Patient S81-252	DOB:	1-Mar-1965	Collected:	27-Oct-2018
Address:	6619-2405 Wesbrook Mall, Vancouver, BC, V6T 1Z3	myDNA ID:	57230	Received:	30-Nov-2018
		Pathology No:	81995N4T3V1	Reported:	6-Dec-2018

Doctor: Dr Corey Nislow
Copy to:

Clinical Notes:

Genetic interpretation by: myDNA
Associate Professor Les Sheffield, MB.BS.
FRACP Approved Pathology Practitioner
23077

REPORT SUMMARY

CURRENT MEDICATIONS OVERVIEW

MEDICATION	GENE(S)	PRESCRIBING CONSIDERATIONS BASED ON myDNA TEST
🟠 **Pantoprazole**	*CYP2C19*	Minor – result should be considered as may affect medication response
🟢 **Fluoxetine**	*CYP2C9*<br>*CYP2D6*	Usual prescribing considerations apply

MEDICATIONS THAT DO NOT HAVE PRESCRIBING CONSIDERATIONS BASED ON myDNA TEST

oestrogen (Premarin Tablets), lamotrigine, trazadone

LEGEND: 🔴 Major prescribing considerations 🟠 Minor prescribing considerations 🟢 Usual prescribing considerations

Detailed pharmacogenomic interpretation and recommendations are provided in the *current medications* section below.

GENETIC TEST RESULTS OVERVIEW

GENE	GENOTYPE	PHENOTYPE	GENE	GENOTYPE	PHENOTYPE
CYP2D6	*1/*2	Normal metaboliser	*CYP3A4*	*1/*1	Normal metaboliser
CYP2C19	*1/*17	Rapid metaboliser	*CYP3A5*	*3/*3	Poor metaboliser
CYP2C9	*1/*1	Normal metaboliser	*SLCO1B1*	TT	Normal Transporter Function
VKORC1	GG	Normal VKORC1 enzyme level	*OPRM1*	AA	Higher opioid sensitivity
CYP1A2	*1A/*1F	Normal metaboliser			

Detailed interpretations of genetic test results are provided in the *pharmacogenomic interpretation* section below.

The following diagram provides the range of enzyme activity predicted by the myDNA test.

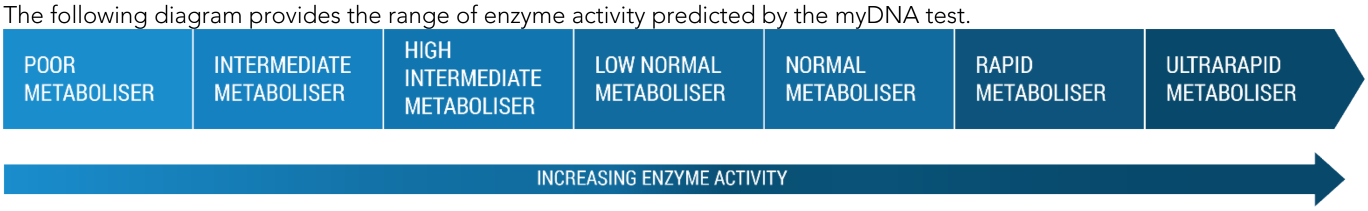

6 December 2018, © 2017 My DNA Life. All rights Reserved.

CURRENT MEDICATIONS

PERSONALISED INTERPRETATION AND RECOMMENDATIONS		
MEDICATION	**INTERPRETATION**	**RECOMMENDATION**
Pantoprazole	*CYP2C19* - Rapid metaboliser:<br>This genotype predicts slightly increased metabolism of pantoprazole which has been linked to an incomplete clinical response in conditions such as oesophagitis and H. pylori.	If response is inadequate, consider 1) a preference for esomeprazole or rabeprazole, 2) increasing the dose, and 3) using divided dosing (i.e. at least twice daily) even of the same overall daily dose.
Fluoxetine	*CYP2D6* - Normal metaboliser<br>*CYP2C9* - Normal metaboliser:<br>The metabolism of fluoxetine is complex due to the involvement of several CYP enzymes (especially CYP2D6 and CYP2C9), the formation of active metabolites and the inhibition of CYP2D6 by fluoxetine and its metabolites.<br><br>The CYP2D6 genotype predicts normal fluoxetine exposure and normal formation of the active S-norfluoxetine metabolite. The CYP2C9 genotype predicts normal metabolism via this pathway. However, fluoxetine and its metabolites can strongly inhibit CYP2D6 function, converting the phenotype to an intermediate or poor metaboliser which can last for up to 9 weeks after cessation of fluoxetine (this is particularly relevant if commencing a drug extensively metabolised by CYP2D6 during this time). This CYP2D6 inhibition is dose and duration of therapy dependent and could potentially lead to late onset adverse effects on a previously tolerated fluoxetine dose.	Standard dosing and prescribing measures apply.<br><br>If adverse effects are a concern, consider an alternative antidepressant for which normal metabolism is predicted.

POTENTIAL DRUG INTERACTIONS

The effect of drug-drug interactions can be additive to the effect of genotype on drug metabolism. Inhibitors can decrease and inducers can increase metabolism, leading to changes in drug concentration and clinical effects.

Comments in the current and future medications sections only consider the effects of the patient's genotype, not those due to interacting drugs. For the health professional's consideration, the table below identifies which of the patient's current drugs may inhibit or induce those enzymes tested by myDNA. The extent of the inhibition or induction depends on the dose and duration of the therapy. The overall effect on metabolism by a specific enzyme may be estimated by considering both the genetic finding and the potential interacting drug.

MEDICATION	INHIBITOR – MODERATE	INHIBITOR - STRONG	INDUCER
Fluoxetine	CYP2C19	CYP2D6	

6 December 2018, © 2017 My DNA Life. All rights Reserved.

myDNA Medication

FUTURE MEDICATIONS

The following tables outline personalised recommendations for future medications.

NOTE: These tables do not account for the effect of any inhibitors or inducers. The table is not an all-inclusive list of medications but includes many commonly prescribed medications.

MEDICATIONS WITH *MAJOR* PRESCRIBING CONSIDERATIONS

DRUG CATEGORY	MEDICATION	GENE(S) INVOLVED	POTENTIAL CLINICAL ISSUES	PUBLISHED GUIDELINES
Antidepressants - SSRIs	Citalopram	*CYP2C19*	Reduced / inadequate response	*CPIC*[1]
	Escitalopram	*CYP2C19*	Reduced / inadequate response	*CPIC*[1]
Antidepressants - tricyclic antidepressants	Amitriptyline	*CYP2D6* *CYP2C19*	Reduced / inadequate response	*CPIC*[2]
	Clomipramine	*CYP2D6* *CYP2C19*	Reduced / inadequate response	*CPIC*[2]
	Doxepin	*CYP2D6* *CYP2C19*	Reduced / inadequate response	*CPIC*[2]
	Imipramine	*CYP2D6* *CYP2C19*	Reduced / inadequate response	*CPIC*[2]
Antifungals - Azoles	Voriconazole	*CYP2C19*	Reduced / inadequate response	*CPIC*[3]

MEDICATIONS WITH *MINOR* PRESCRIBING CONSIDERATIONS

DRUG CATEGORY	MEDICATION	GENE(S) INVOLVED	POTENTIAL CLINICAL ISSUES	PUBLISHED GUIDELINES
Antidepressants - other	Moclobemide	*CYP2C19*	Reduced / inadequate response	-
Antidepressants - SSRIs	Sertraline	*CYP2C19*	Reduced / inadequate response	*CPIC*[1]
Antidiabetics	Gliclazide	*CYP2C9* *CYP2C19*	Reduced / inadequate response	-
Antiplatelet drugs	Clopidogrel	*CYP2C19*	Adverse effects	*CPIC*[4]
Benzodiazepines	Clobazam	*CYP2C19*	Reduced / inadequate response	-
	Diazepam	*CYP2C19*	Reduced / inadequate response	-
Miscellaneous	Cyclophosphamide	*CYP2C19*	Increased therapeutic and/or adverse effects	-
	Naltrexone	*OPRM1*	Associated with reduced response to naltrexone	
	Proguanil	*CYP2C19*	Altered response	-
Opioid Analgesics	Morphine	*OPRM1*	Associated with increased therapeutic and/or adverse effects to morphine	-
Proton pump inhibitors	Dexlansoprazole	*CYP2C19*	Reduced / inadequate response	-
	Esomeprazole	*CYP2C19*	Reduced / inadequate response	-
	Lansoprazole	*CYP2C19*	Reduced / inadequate response	-
	Omeprazole	*CYP2C19*	Reduced / inadequate response	-
	Pantoprazole	*CYP2C19*	Reduced / inadequate response	-
	Rabeprazole	*CYP2C19*	Reduced / inadequate response	-

MEDICATIONS WITH *USUAL* PRESCRIBING CONSIDERATIONS

DRUG CATEGORY	MEDICATION	GENE(S) INVOLVED	POTENTIAL CLINICAL ISSUES	PUBLISHED GUIDELINES
ADHD - miscellaneous agents	Atomoxetine	*CYP2D6*	No altered effect predicted by genotype	-

6 December 2018, © 2017 My DNA Life. All rights Reserved.

MEDICATIONS WITH *USUAL* PRESCRIBING CONSIDERATIONS

DRUG CATEGORY	MEDICATION	GENE(S) INVOLVED	POTENTIAL CLINICAL ISSUES	PUBLISHED GUIDELINES
Angiotensin receptor blockers	Irbesartan	*CYP2C9*	No altered effect predicted by genotype	-
	Losartan	*CYP2C9*	No altered effect predicted by genotype	-
Antiarrhythmics	Flecainide	*CYP2D6*	No altered effect predicted by genotype	-
	Propafenone	*CYP2D6*	No altered effect predicted by genotype	-
Anticholinergics (genitourinary)	Darifenacin	*CYP2D6*	No altered effect predicted by genotype	-
	Fesoterodine	*CYP2D6*	No altered effect predicted by genotype	-
	Tolterodine	*CYP2D6*	No altered effect predicted by genotype	-
Anticholinesterases	Donepezil	*CYP2D6*	No altered effect predicted by genotype	-
	Galantamine	*CYP2D6*	No altered effect predicted by genotype	-
Anticoagulants	Acenocoumarol	*VKORC1* *CYP2C9*	Normal acenocoumarol sensitivity	
	Warfarin	*VKORC1* *CYP2C9*	Normal warfarin sensitivity	*FDA*[5]
Antidepressants - other	Mirtazapine	*CYP2D6* *CYP1A2*	No altered effect predicted by genotype	-
	Vortioxetine	*CYP2D6*	No altered effect predicted by genotype	-
Antidepressants - serotonin noradrenaline reuptake inhibitors	Duloxetine	*CYP2D6* *CYP1A2*	No altered effect predicted by genotype	-
	Venlafaxine	*CYP2D6*	No altered effect predicted by genotype	*DPWG*[6]
Antidepressants - SSRIs	Fluoxetine	*CYP2D6* *CYP2C9*	No altered effect predicted by genotype	-
	Fluvoxamine	*CYP2D6* *CYP1A2*	No altered effect predicted by genotype	*CPIC*[1]
	Paroxetine	*CYP2D6*	No altered effect predicted by genotype	*CPIC*[1]
Antidepressants - tricyclic antidepressants	Desipramine	*CYP2D6*	No altered effect predicted by genotype	*CPIC*[2]
	Nortriptyline	*CYP2D6*	No altered effect predicted by genotype	*CPIC*[2]
Antidiabetics	Glimepiride	*CYP2C9*	No altered effect predicted by genotype	-
	Glyburide	*CYP2C9*	No altered effect predicted by genotype	-
Antiemetics	Metoclopramide	*CYP2D6*	No altered effect predicted by genotype	-
	Ondansetron	*CYP2D6*	No altered effect predicted by genotype	*CPIC*[7]
Antiepileptics	Phenytoin	*CYP2C9*	No altered effect predicted by genotype	*CPIC*[8]
Antihistamines	Chlorpheniramine	*CYP2D6*	No altered effect predicted by genotype	-
	Dexchlorpheniramine	*CYP2D6*	No altered effect predicted by genotype	-

6 December 2018, © 2017 My DNA Life. All rights Reserved.

1-844-472-7896 · www.mydna.life

MEDICATIONS WITH *USUAL* PRESCRIBING CONSIDERATIONS				
DRUG CATEGORY	MEDICATION	GENE(S) INVOLVED	POTENTIAL CLINICAL ISSUES	PUBLISHED GUIDELINES
	Promethazine	*CYP2D6*	No altered effect predicted by genotype	-
Antipsychotics	Aripiprazole	*CYP2D6*	No altered effect predicted by genotype	-
	Brexpiprazole	*CYP2D6*	No altered effect predicted by genotype	-
	Chlorpromazine	*CYP2D6*	No altered effect predicted by genotype	-
	Clozapine	*CYP1A2*	No altered effect predicted by genotype	-
	Haloperidol	*CYP2D6*	No altered effect predicted by genotype	-
	Olanzapine	*CYP1A2*	No altered effect predicted by genotype	-
	Pimozide	*CYP2D6*	No altered effect predicted by genotype	
	Quetiapine	*CYP3A4*	No altered effect predicted by genotype	-
	Risperidone	*CYP2D6*	No altered effect predicted by genotype	-
	Zuclopenthixol	*CYP2D6*	No altered effect predicted by genotype	-
Antitussives	Dextromethorphan	*CYP2D6*	No altered effect predicted by genotype	-
Beta blockers	Carvedilol	*CYP2D6*	No altered effect predicted by genotype	-
	Metoprolol	*CYP2D6*	No altered effect predicted by genotype	-
	Nebivolol	*CYP2D6*	No altered effect predicted by genotype	-
	Propranolol	*CYP2D6* *CYP1A2*	No altered effect predicted by genotype	-
Calcineurin inhibitors	Tacrolimus	*CYP3A5*	No altered effect predicted by genotype	*CPIC*[9]
Glaucoma - ocular preparations	Timolol	*CYP2D6*	No altered effect predicted by genotype	-
Hypnotics	Melatonin	*CYP1A2*	No altered effect predicted by genotype	-
Immunomodulators and antineoplastics	Tamoxifen	*CYP2D6*	No altered effect predicted by genotype	*CPIC*[10]
Miscellaneous	Atazanavir	*CYP3A5*	No altered effect predicted by genotype	-
	Eliglustat	*CYP2D6*	No altered effect predicted by genotype	*TGA*[11]
Neurological drugs	Tetrabenazine	*CYP2D6*	No altered effect predicted by genotype	*FDA*[12]
NSAIDs	Celecoxib	*CYP2C9*	No altered effect predicted by genotype	-
	Diclofenac	*CYP2C9*	No altered effect predicted by genotype	-
	Flurbiprofen	*CYP2C9*	No altered effect predicted by genotype	-
	Ibuprofen	*CYP2C9*	No altered effect predicted by genotype	-

6 December 2018, © 2017 My DNA Life. All rights Reserved.

MEDICATIONS WITH *USUAL* PRESCRIBING CONSIDERATIONS				
DRUG CATEGORY	MEDICATION	GENE(S) INVOLVED	POTENTIAL CLINICAL ISSUES	PUBLISHED GUIDELINES
	Indomethacin	*CYP2C9*	No altered effect predicted by genotype	-
	Mefenamic Acid	*CYP2C9*	No altered effect predicted by genotype	-
	Meloxicam	*CYP2C9*	No altered effect predicted by genotype	-
	Piroxicam	*CYP2C9*	No altered effect predicted by genotype	-
Opioid Analgesics	Codeine	*CYP2D6* *OPRM1*	Associated with increased sensitivity to codeine	*CPIC*[13]
	Hydrocodone	*CYP2D6*	No altered effect predicted by genotype	
	Oxycodone	*CYP2D6*	No altered effect predicted by genotype	-
	Tramadol	*CYP2D6*	No altered effect predicted by genotype	-
Psychostimulants	Dextroamphetamine	*CYP2D6*	No altered effect predicted by genotype	-
	Lisdexamfetamine	*CYP2D6*	No altered effect predicted by genotype	-
Statins	Atorvastatin	*SLCO1B1* *CYP3A4*	No altered effect predicted by genotype	-
	Fluvastatin	*SLCO1B1* *CYP2C9*	No altered effect predicted by genotype	-
	Pravastatin	*SLCO1B1*	No altered effect predicted by genotype	-
	Rosuvastatin	*SLCO1B1*	No altered effect predicted by genotype	-
	Simvastatin	*SLCO1B1* *CYP3A4*	No altered effect predicted by genotype	*CPIC*[14]

LEGEND:

CPIC = Clinical Pharmacogenetics Implementation Consortium
DPWG = The Royal Dutch Pharmacists Association – Pharmacogenetics Working Group
TGA = Therapeutic Goods Administration (Australia)
FDA = Food and Drug Administration (US)

CPIC and DPWG guidelines are available on the PharmGKB website www.pharmgkb.org/view/dosing-guidelines.do

PHARMACOGENOMIC INTERPRETATION

EXPLANATION OF GENETIC RESULTS		
GENE	GENOTYPE	PREDICTED FUNCTION
CYP2D6	*1/*2	*CYP2D6* - Normal metaboliser<br>Due to the presence of two normal function alleles, this individual is predicted to have a normal metaboliser phenotype. For a drug extensively metabolised by CYP2D6, drug exposure and clinical effects may be expected to lie within the normal range.

6 December 2018, © 2017 My DNA Life. All rights Reserved.

EXPLANATION OF GENETIC RESULTS		
GENE	GENOTYPE	PREDICTED FUNCTION
CYP2C19	*1/*17	*CYP2C19* - Rapid metaboliser<br>Due to the presence of one normal function allele and one increased function allele, this individual is predicted to have a rapid metaboliser phenotype. For a drug extensively metabolised by CYP2C19, drug exposure and clinical effects may either be slightly decreased (for an active drug) or slightly increased (for a prodrug). This individual is at risk of therapeutic failure (active drug) or adverse effects (prodrug).
CYP2C9	*1/*1	*CYP2C9* - Normal metaboliser<br>Due to the presence of two normal function alleles, this individual is predicted to have a normal metaboliser phenotype. For a drug extensively metabolised by CYP2C9, drug exposure and clinical effects may be expected to lie within the normal range.
VKORC1	GG	*VKORC1* - Normal VKORC1 enzyme level<br>The VKORC1 enzyme is predicted to be present in normal amounts and the response to warfarin will be normal. The *CYP2C9* genotype should also be considered together with the *VKORC1* genotype for calculating the initial warfarin dose.
CYP1A2	*1A/*1F	*CYP1A2* - Normal metaboliser<br>Due to the presence of only one copy of the *1F allele, this individual is predicted to have a normal metaboliser phenotype. Normal metabolism of CYP1A2 substrate drugs is predicted. Furthermore, metabolism is not expected to be increased by exposure to inducers such as tobacco smoking and certain dietary components and drugs.
CYP3A4	*1/*1	*CYP3A4* - Normal metaboliser<br>The *22 allele is not present and this individual is expected to have a normal metaboliser phenotype. Whilst many drugs are known to be metabolised by CYP3A4, relatively few genetic variations have been found that affect metabolism of a limited number of these drugs.
CYP3A5	*3/*3	*CYP3A5* - Poor metaboliser<br>Due to the presence of two no function alleles, this individual is predicted to have a poor metaboliser phenotype (CYP3A5 non-expresser). CYP3A5 is known to metabolise certain drugs, including tacrolimus. Note that this individual's genotype is the most common one amongst Caucasians.
SLCO1B1	TT	*SLCO1B1* - Normal Transporter Function<br>The decreased function *5 allele is not present and this individual is predicted to have normal function of the *SLCO1B1* encoded transporter. The transporter is important for the clearance of certain drugs, including simvastatin.
OPRM1	AA	*OPRM1* - Higher opioid sensitivity<br>The AA genotype contains two normal alleles for the OPRM1 gene which encodes the mu opioid receptor. Whilst the evidence around OPRM1 genetic variation continues to develop, it appears that this result is associated with increased sensitivity to certain opioids (in particular, morphine) compared to those with the variant allele (G). These findings are supported by a number of cohort studies and at least two meta-analyses[15,16] however, this is not shown in all studies. For naltrexone in the management of alcohol use disorder, some studies have shown an association of this result with a reduced response compared to those with the variant allele. Note the frequency of the variant allele (G) is higher in people of Asian ancestry (around 40%) than European ancestry (around 15%).

REFERENCES

1. Hicks J, Bishop J, Sangkuhl K, Müller D, Ji Y, Leckband S et al. Clinical Pharmacogenetics Implementation Consortium (CPIC) Guideline for CYP2D6 and CYP2C19 Genotypes and Dosing of Selective Serotonin Reuptake Inhibitors. Clinical Pharmacology & Therapeutics. 2015;98(2):127-134.

6 December 2018, © 2017 My DNA Life. All rights Reserved.

2. Hicks JK, Sangkuhl K, Swen JJ, Ellingrod VL, Muller DJ, Shimoda K, et al. Clinical pharmacogenetics implementation consortium guideline (CPIC) for CYP2D6 and CYP2C19 genotypes and dosing of tricyclic antidepressants: 2016 update. Clin Pharmacol Ther. 2016.

3. Moriyama B, Obeng A, Barbarino J, Penzak S, Henning S, Scott S et al. Clinical Pharmacogenetics Implementation Consortium (CPIC®) Guideline for CYP2C19 and Voriconazole Therapy. Clinical Pharmacology & Therapeutics. 2016;.

4. Scott S, Sangkuhl K, Stein C, Hulot J, Mega J, Roden D et al. Clinical Pharmacogenetics Implementation Consortium Guidelines for CYP2C19 Genotype and Clopidogrel Therapy: 2013 Update. Clin Pharmacol Ther. 2013;94(3):317-323.

5. DailyMed - COUMADIN- warfarin sodium tablet . 2016. DailyMed - COUMADIN- warfarin sodium tablet . [ONLINE] Available at: https://dailymed.nlm.nih.gov/dailymed/drugInfo.cfm?setid=d91934a0-902e-c26c-23ca-d5accc4151b6#_Refs12.5. [Accessed 11 October 2016].

6. Swen J, Nijenhuis M, de Boer A, Grandia L, Maitland-van der Zee A, Mulder H et al. Pharmacogenetics: From Bench to Byte— An Update of Guidelines. Clin Pharmacol Ther. 2011;89(5):662-673.

7. Bell G, Caudle K, Whirl-Carrillo M, Gordon R, Hikino K, Prows C et al. Clinical Pharmacogenetics Implementation Consortium (CPIC) guideline for CYP2D6 genotype and use of ondansetron and tropisetron. Clinical Pharmacology & Therapeutics. 2017 (epub ahead of print).

8. Caudle KE, Rettie AE, Whirl-Carrillo M, Smith LH, Mintzer S, Lee MTM et al. Clinical Pharmacogenetics Implementation Consortium Guidelines for CYP2C9 and HLA-B Genotypes and Phenytoin Dosing. Clin Pharmacol Ther. 2014;95(5):542-548

9. Birdwell K, Decker B, Barbarino J, Peterson J, Stein C, Sadee W et al. Clinical Pharmacogenetics Implementation Consortium (CPIC) Guidelines for CYP3A5 Genotype and Tacrolimus Dosing. Clin Pharmacol Ther. 2015;98(1):19-24.

10. Goetz MP, Sangkuhl K, Guchelaar HJ, Schwab M, Province M, Whirl-Carrillo M, et al. Clinical Pharmacogenetics Implementation Consortium (CPIC) Guideline for CYP2D6 and Tamoxifen Therapy. Clin Pharmacol Ther. 2018.

11. TGA eBS - Product and Consumer Medicine Information Licence. 2016. TGA eBS - Product and Consumer Medicine Information Licence. [ONLINE] Available at:https://www.ebs.tga.gov.au/ebs/picmi/picmirepository.nsf/pdf?OpenAgent&id=CP-2015-PI-01366-1. [Accessed 11 October 2016].

12. DailyMed - TETRABENAZINE- tetrabenazine tablet. 2017. [ONLINE] Available at: https://dailymed.nlm.nih.gov/dailymed/drugInfo.cfm?setid=a9c0e69d-adb2-4fca-9410-c9ae9ccf93ee#section-8.7 [Accessed 25 November 2017]

13. Crews, K R et al. Clinical Pharmacogenetics Implementation Consortium Guidelines For Cytochrome P450 2D6 Genotype And Codeine Therapy: 2014 Update. Clin Pharmacol Ther 95.4 (2014): 376-382.

14. Ramsey LB, Johnson SG, Caudle KE, Haidar CE, Voora D, Wilke RA, et al. The Clinical Pharmacogenetics Implementation Consortium Guidelines for SLCO1B1 and Simvastatin-Induced Myopathy: 2014 Update. Clin Pharmacol Ther. Online publication 9 July 2014. doi:10.1038/clpt.2014.125

15. Zhen-Yu Ren, Xiao-Qing Xu, Yan-Ping Bao, Jia He, Le Shi et al. The Impact of Genetic Variation on Sensitivity to Opioid Analgesics in Patients with Postoperative Pain: A Systematic Review and Meta-Analysis. Pain Physician 2015; 18:131-152.

16. In Cheol Hwang, Ji-Young Park, Seung-Kwon Myung, Hong Yup Ahn, Ken-ichi Fukuda, Qin Liao. OPRM1 A118G Gene Variant and Postoperative Opioid Requirement A Systematic Review and Meta-analysis. Anesthesiology 2014; 121:825-34.

6 December 2018, © 2017 My DNA Life. All rights Reserved.

REPORT PREPARED BY:

Associate Professor Les Sheffield
Approved Pathology Practitioner
MB.BS. FRACP

Sam Mostafa
Consultant Pharmacist
BPharm, MPS, AACPA

Prepared by: My DNA Life Australia Pty Ltd. - A/Prof Les Sheffield, Accredited Pathology Practitioner
Laboratory Results provided by: myDNA

Disclaimer: The pharmacogenomic test result in this report is just one factor that the prescribing doctor will take into consideration when determining a patient's appropriate medication and dose. These interpretations are being provided to the prescribing doctor as a tool to assist in the prescription of medication. Patients are advised not to alter the dose or stop any medications unless instructed by the doctor. The interpretation and clinical recommendations are based on the above results as reported by myDNA and its affiliates and also uses information provided to myDNA by the referring doctor. This report also assumes correct labelling of sample tubes and that the sample is from the above patient.

MYDNA CLINICAL SUPPORT

For all health practitioner enquiries please contact myDNA clinical support

T: 1-844-472-7896

E: clinical@mydna.life
